# Can the Clinical Frailty Scale on admission predict 30-day survival, postoperative complications, and institutionalization in patients with fragility hip fracture? a cohort study of 1,255 patients

**DOI:** 10.1302/0301-620X.104B8.BJJ-2020-1835.R2

**Published:** 2022-08-01

**Authors:** Adeel Ikram, Alan R. Norrish, Ben A. Marson, Simon Craxford, John R. F. Gladman, Ben J. Ollivere

**Affiliations:** 1 Nottingham University Hospitals NHS Trust, Nottingham, UK; 2 University of Nottingham, Nottingham, UK; 3 Queen Elizabeth Hospital, King’s Lynn, UK; 4 NIHR Nottingham Biomedical Research Unit, Nottingham, UK; 5 NIHR Applied Research Collaboration East Midlands, Nottingham, UK

**Keywords:** Frailty score, Hip fracture, Mortality, Complications, Institutionalization, Outcomes, Frailty, Survival, Clinical Frailty Scale, fracture of the hip, postoperative complications, frailty, Clinicians, Nottingham Hip Fracture Score, orthogeriatrician, comorbidities, fractured neck of femur, trauma

## Abstract

**Aims:**

We assessed the value of the Clinical Frailty Scale (CFS) in the prediction of adverse outcome after hip fracture.

**Methods:**

Of 1,577 consecutive patients aged > 65 years with a fragility hip fracture admitted to one institution, for whom there were complete data, 1,255 (72%) were studied. Clinicians assigned CFS scores on admission. Audit personnel routinely prospectively completed the Standardised Audit of Hip Fracture in Europe form, including the following outcomes: 30-day survival; in-hospital complications; length of acute hospital stay; and new institutionalization. The relationship between the CFS scores and outcomes was examined graphically and the visual interpretations were tested statistically. The predictive values of the CFS and Nottingham Hip Fracture Score (NHFS) to predict 30-day mortality were compared using receiver operating characteristic area under the curve (AUC) analysis.

**Results:**

Significant non-linear associations between CFS and outcomes were observed. Risk of death within 30 days rose linearly for CFS 1 to 5, but plateaued for CFS > 5. The incidence of complications and length of stay rose linearly for CFS 1 to 4, but plateaued for CFS > 4. In contrast, the risk of new institutionalization rose linearly for CFS 1 to 8. The AUCs for 30-day mortality for the CFS and NHFS were very similar: CFS AUC 0.63 (95% CI 0.57 to 0.69) and NHFS AUC 0.63 (95% CI 0.57 to 0.69).

**Conclusion:**

Use of the CFS may provide useful information on outcomes for fitter patients presenting with hip fracture, but completion of the CFS by the admitting orthopaedic team does not appear successful in distinguishing between higher CFS categories, which define patients with frailty. This makes a strong case for the role of the orthogeriatrician in the early assessment of these patients. Further work is needed to understand why patients assessed as being of mild, moderate, and severe frailty do not result in different outcomes.

Cite this article: *Bone Joint J* 2022;104-B(8):980–986.

## Introduction

The incidence of fragility hip fractures is increasing.^1^ These are caused by low-energy trauma, usually resulting from a fall from standing height in older people.^2^ Overall, the outcomes of hip fracture are poor: according to statistics from the National Hip Fracture Database in 2019, 6.5% of patients died within a month,^3^ 11% to 15% of patients are institutionalized at six months,^4^ and 20% develop postoperative complications.^5^ It would be useful for clinicians, patients, and their families when making care plans to be able to distinguish between those at high and those at low risk of a poor outcome; frailty status may offer the opportunity to do so. Frailty is a health state related to the ageing process where multiple body systems gradually lose their inbuilt reserves: older people who are frail are likely to decompensate in the face of an external challenge, whereas those who are robust are more resilient to such challenges. Two studies have demonstrated that frailty indices are predictive of short-^6^ and long-term mortality^7^ and hospital length of stay (LOS)^6^ following hip fracture, but neither study examined whether frailty measures predict other clinically important outcomes associated with hip fracture, such as complications or institutionalization. In recent years, particularly in the UK due to the efforts of the Acute Frailty Network,^8^ there has been widespread adoption of the Clinical Frailty Scale (CFS) in all older patients admitted to hospital. The CFS is an assessment of a patient’s frailty status based on the two weeks prior to their admission, and is only validated in those above the age of 65.^9^ It is completed following a comprehensive history and examination, and verified with a collateral history where appropriate. It is cross-referenced with observations of other healthcare professionals. Along with functional assessments, the ability to complete higher-order tasks (e.g. cooking and maintaining self-care) is also obtained to allocate the appropriate CFS score.

The objectives of this study in patients with hip fracture were to determine the relationship between CFS and 30-day mortality, inpatient complication rate, institutionalization, and length of hospital stay, and to compare the predictive value of the CFS with that of the Nottingham Hip Fracture Score (NHFS).^10^

## Methods

This was a prospective observational cohort study. All patients presenting with a fragility hip fracture to our institution in the specified time period were eligible for inclusion. All patients without a recorded CFS categorization and patients aged younger than 65 years were excluded. Patients were identified following referral from the emergency department with a confirmed diagnosis of a hip fracture following low-energy trauma, a fall, or inability to mobilize. Patients presented to our institution, a 1,700-bed hospital group which includes a regional major trauma centre, between the dates of 1 April 2018 and 31 December 2019. Patient data were collected on admission and during inpatient stay in a consecutive series of patients. Our institution collects data on all hip fracture patients using the Standardised Audit of Hip Fracture in Europe (SAHFE) collection form.^11^ Data were collected independently by trained audit staff who administer the local database according to national data protection standards. Research Ethics Committee approval and informed consent were not required, as this study was conducted under existing clinical governance procedures using routine data for quality improvement. All data remained anonymous. Our index test was the CFS,^9^ which is a nine-point scale based on a cumulative deficit model. As the number of deficits a person experiences increases, the level of frailty also increases (Table I). CFS scores were assigned by doctors (specialist registrars or core surgical trainees) trained in the management of hip fracture and frailty.

**Table I.** The Clinical Frailty Scale, adapted from Rockwood et al.^9^

Category	Descriptor	Features
1	Very fit	Robust, active, energetic, motivated. Commonly exercise regularly. Among fittest for age.
2	Well	No active disease symptoms, but less fit than category 1, with less regular exercise or only seasonally very active.
3	Managing well	Medical problems well controlled, not regularly active beyond routine walking.
4	Vulnerable	Not dependent on daily help. Symptoms often limit activities. Feel ‘slowed-up’ or tired during the day.
5	Mildly frail	More evident slowing, need help in high-order ADLs. Typically, impaired shopping, walking alone outside, meal preparation, and housework.
6	Moderately frail	Need help with all outside activities and keeping house. Difficulties with stairs, bathing and, to a lesser extent, dressing.
7	Severely frail	Completely dependent on others for personal care (from either physical or cognitive cause).
8	Very severely frail	Completely dependent and approaching the end of life.
9	Terminally ill	< 6 months life expectancy, but who are not otherwise evidently frail.

ADLs, activities of daily living.

Our reference standard was the NHFS, which is a validated measure of predicting 30-day mortality in patients with hip fracture, developed in our centre. This tool is routinely used at our institution for all hip fracture patients to predict a patient’s risk of death based upon specific parameters (Supplementary Table i). The raw score is calculated through a scoring matrix and then converted to a percentage risk (Supplementary Table ii).

Following a comprehensive history, examination, and discussion with other healthcare professionals, the on-call orthopaedic team assigned a CFS on admission for each patient. The NHFS was also calculated based on admission parameters and patient history to provide a percentage risk of 30-day mortality. Where information was not available, a collateral history was taken from relatives, institutions (nursing/residential care home), general practitioners, previous admission notes, and clinic letters.

Our institution followed a strict ‘fractured neck of femur care pathway’ which standardized care on admission and during inpatient stay. Patients were managed according to evidence-based, national best practice guidelines.^3^ This included light, skilled anaesthesia, early surgery, and an orthogeriatric package of care and rehabilitation. As part of the guidelines, data were collected including demographic characteristics (age, sex, height, and weight), Abbreviated Mental Test scores (AMTS),^12^ mobility status (freely mobile, mobilizes with one aid, mobilizes with two aids or a frame, some indoor mobility but no outdoor mobility, and no functional mobility), NHFS, admission blood results (including full blood count, urea, and electrolytes), type of hip fracture (intracapsular displaced and undisplaced, intertrochanteric, and reverse oblique/subtrochanteric), and frailty as demonstrated by CFS categorization. Patient comorbidity data were collected, which included the following conditions: atrial fibrillation; asthma; anaemia on admission; blindness; chronic obstructive pulmonary disease; chronic kidney disease; cerebrovascular accident/transient ischaemic attack; dementia/cognitive impairment; diabetes; dysphagia; depression; alcohol excess; epilepsy; gastroesophageal reflux disease; heart failure; hypertension; ischaemic heart disease; liver disease; malignancy; obesity; pacemaker; Parkinson’s disease; rheumatoid arthritis; psychosis; and smoking status. Patient procedural data collected included mechanism of injury; date and time of admission; date, time, and type of surgery; length of hospital stay; and, where appropriate, the date of death. Data were collected on in-hospital postoperative complications including pulmonary embolus, pneumonia, urinary tract infection, cerebrovascular accident, myocardial infarction/acute coronary syndrome, deep vein thrombosis, dislocation, failure of fixation, blood transfusion, acute kidney injury, pressure ulcer, *Clostridium difficile* infection, and reoperation in 120 days. We collected 30-day mortality data, inpatient postoperative complications, LOS, and discharge information (admission to residential care/nursing care).

The CFS and NHFS were recorded on admission. Missing NHFS scores were calculated retrospectively. We feel this was acceptable, since the data needed to perform the calculation were collected prospectively. CFS scores were not calculated retrospectively as it is a measure which may be subject to recall bias. Therefore, patients with no recorded CFS were excluded from the study.

### Statistical analysis

Statistical analysis of the data was performed with SPSS Statistics for Mac v. 26 (IBM, USA). At the outset of analysis, we have assumed no a priori relationship between CFS and our choice of outcomes. Our first means of examining the relationship between the CFS and outcomes of interest was visual inspection of the data through bar graphs. Based on this visual inspection, we then chose the appropriate statistical tests. Tests included chi-squared test for categorical variables, analysis of variance for continuous variables (ANOVA), and two-tailed Spearman’s correlation test between own home to institution, and CFS. Receiver operating characteristic (ROC) curve and Kaplan-Meier survival analyses were performed.

## Results

In all, 1,577 patients with hip fracture were identified between 1 April 2018 and 31 December 2019. Of these, 172 patients were excluded from analysis as they were aged < 65 years, and CFS scores were missing for 150. Ultimately, 1,255 patients (72%) were included in the analysis. Baseline demographic and clinical details, stratified by CFS, are given in Table II. As anticipated, baseline variables associated with frailty (age, cognitive impairment, abnormal BMI, residential status, mobility, disability, and comorbidity) were all increasingly prevalent with increasing CFS (Table II). Very few patients scored CFS 8 (n = 14) or 9 (n = 2).

**Table II.** Patient demographic data, pre-admission residence, functional status, and number of comorbidities categorized by Clinical Frailty Scale.

Characteristic	CFS								
	1	2	3	4	5	6	7	8	9
Patients, n (%)	37 (2.9)	112 (8.9)	222 (17.7)	236 (18.8)	227 (18.1)	249 (19.8)	156 (12.4)	14 (1.1)	2 (0.2)
Male, n	9	37	76	76	63	60	39	3	2
Female (%)	28 (75.7)	75 (67.0)	146 (65.8)	160 (67.8)	164 (72.2)	189 (75.9)	117 (75.0)	11 (78.6)	0 (0.0)
Mean age (95% CI)	76.95 (74.24 to 79.65)	77.18 (75.56 to 78.60)	81.80 (80.90 to 82.70)	85.53 (82.57 to 84.50)	84.84 (83.82 to 85.86)	86.47 (85.60 to 87.35)	86.57 (85.45 to 87.69)	88.36 (86.05 to 90.66)	85.0 (-16.65 to 186.65)
Mean AMTS (95% CI)	9.76 (9.57 to 9.94)	9.32 (9.02 to 9.61)	8.73 (8.46 to 9.00)	8.02 (7.67 to 8.37)	7.05 (6.64 to 7.45)	4.86 (4.41 to 5.32)	2.31 (0.31 to 3.69)	2.0 (0.31 to 3.69)	4.5 (-52.68 to 61.88)
Mean NHFS (95% CI)	3.89 (3.55 to 4.23)	4.05 (3.86 to 4.24)	4.52 (4.36 to 4.67)	4.86 (4.71 to 5.02)	5.23 (5.08 to 5.39)	5.67 (5.53 to 5.82)	6.03 (5.85 to 6.20)	6.29 (5.43 to 7.15)	8.0 (-4.71 to 20.71)
Underweight, n (%)	5 (14.3)	5 (5.0)	14 (6.9)	21 (9.8)	30 (15.2)	33 (15.9)	23 (19.3)	2 (20.0)	0 (0.0)
Overweight, n (%)	8 (22.9)	40 (39.6)	67 (32.8)	80 (37.2)	62 (31.5)	52 (25.1)	18 (15.1)	1 (10.0)	0 (0.0)
Under/overweight, n (%)	13 (37.1)	45 (44.6)	81 (39.7)	101 (47.0)	92 (46.7)	85 (41.1)	41 (34.5)	3 (30.0)	0 (0.0)
**Pre-admission residence, n**									
Own home	37	111	217	223	205	183	87	6	2
Residential care	0	1	3	8	16	40	43	2	0
Nursing home	0	0	2	5	6	26	26	6	0
**Pre-admission mobility, n (%)**									
Freely mobile,	35 (94.6)	97 (86.6)	141 (63.5)	73 (30.9)	44 (19.4)	34 (13.7)	30 (19.2)	2 (14.3)	0 (0.0)
Mobile with 1 aid	2 (5.4)	13 (11.6)	64 (28.8)	114 (48.3)	75 (33.0)	49 (19.7)	21 (13.5)	3 (21.4)	0 (0.0)
Mobile with 2 aids/frame	0 (0.0)	1 (0.9)	15 (6.8)	42 (17.8)	95 (41.9)	118 (47.4)	56 (35.9)	3 (21.4)	1 (50.0)
Some indoor, no outdoor mobility	0 (0.0)	1 (0.9)	2 (0.9)	5 (2.1)	10 (4.4)	42 (16.9)	37 (23.7)	3 (21.4)	0 (0.0)
No functional mobility	0 (0.0)	0 (0.0)	0 (0.0)	0 (0.0)	1 (0.4)	1 (0.4)	8 (5.1)	0 (0.0)	1 (50.0)
Need assistance with ADLs	0 (0.0)	3 (2.7)	13 (5.9)	39 (16.5)	61 (26.9)	90 (36.1)	84 (53.8)	6 (42.9)	1 (50.0)
Mean comorbidities per patient, n (95% CI)	1.19 (0.85 to 1.53)	1.54 (1.30 to 1.79)	2.10 (1.89 to 2.31)	2.43 (2.19 to 2.66)	3.07 (2.84 to 3.30)	3.20 (2.99 to 3.41)	3.35 (3.08 to 3.61)	3.79 (2.58 to 4.99)	1.50 (-4.85 to 7.85)

ADLs, activities of daily living; AMTS, Abbreviated Mental Test Score; CFS, Clinical Frailty Scale; CI, confidence interval; NHFS, Nottingham Hip Fracture Score.

**Fig. 1:**
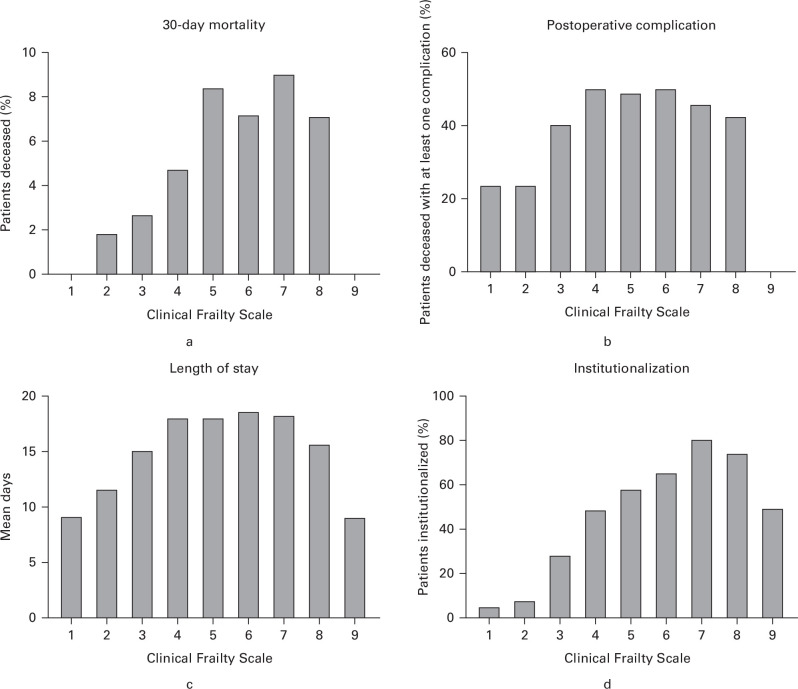
a) Clinical Frailty Scale (CFS) and 30-day mortality outcome. b) CFS and development of at least one postoperative complication. c) CFS and acute inpatient length of stay. d) CFS and new institutionalization.

Table III demonstrates the outcomes of interest according to admission CFS, and Figure 1 illustrates these results graphically. Figure 1 shows that the exact relationship between CFS and each outcome differed, and suggests that 30-day mortality increased linearly from CFS 1 to 5, but was stable from CFS 5 to 8. Statistical testing confirmed a significant positive linear relationship between CFS 1 to 5 and 30-day mortality (Spearman’s Rho 0.12; p < 0.001) and no significant relationship between CFS 5 to 9 and 30-day mortality (rs(646) = 0.00; p = 0.980). Visual inspection suggested that the incidence of postoperative complications similarly rose linearly from CFS 1 to 4, but was stable from CFS 4 to 8. Statistical testing confirmed a significant positive linear relationship between CFS 1 to 4 and presence of postoperative complications (rs(605) = 0.21; p < 0.001), and no significant relationship between CFS 4 to 9 and postoperative complications (rs(882) = -0.03; p = 0.376). Visual inspection suggested that LOS rose linearly from CFS 1 to 4, but was stable from CFS 4 to 8. Statistical testing confirmed a significant positive linear relationship between CFS 1 to 4 and LOS (rs(589) = 0.31; p < 0.001), and no significant relationship between CFS 4 to 9 and LOS (rs(857) = -0.18; p = 0.594). Visual inspection suggested that the incidence of institutionalization rose linearly from CFS 1 to 7. Statistical testing confirmed a significant positive linear relationship between CFS 1 to 7 and institutionalization (rs(935) = 0.43; p < 0.001), and no significant relationship between CFS 7 to 9 and institutionalization (rs(77) = -0.10; p = 0.399).

**Table III.** Frailty outcomes, categorized by Clinical Frailty Scale.

Outcome	CFS									
	1	2	3	4	5	6	7	8	9	p-value
Patients, n	37	112	222	236	227	249	156	14	2	
30-day mortality, n (%)	0 (0.0)	2 (1.8)	6 (2.7)	11 (4.7)	19 (8.4)	18 (7.2)	14 (9.0)	1 (7.1)	0 (0.0)	< 0.001*
Presence of complications, n (%)	8 (21.6)	27 (24.1)	91 (41.0)	120 (50.8)	112 (49.3)	126 (50.6)	72 (46.2)	6 (42.9)	0 (0.0)	< 0.001*
Own home to institution, n (%)	2 (5.6)	9 (8.6)	56 (28.7)	100 (49.3)	99 (57.9)	102 (66.2)	59 (80.8)	3 (75.0)	1 (50.0)	0.427†
Own home to own home, n (%)	34 (91.9)	96 (86.5)	139 (64.1)	103 (46.2)	72 (35.1)	52 (28.4)	14 (16.1)	1 (16.7)	1 (50.0)	< 0.001*
Mean length of stay, days (95% CI)	9.03 (7.81 to 10.25)	11.69 (10.67 to 12.72)	15.17 (14.04 to 16.30)	18.11 (16.51 to 19.70)	18.12 (16.40 to 19.85)	18.64 (16.92 to 20.35)	18.28 (15.99 to 20.58)	15.69 (10.08 to 21.30)	9.00 (-3.71 to 21.71)	< 0.001‡

* Chi-squared test.

† Spearman's correlation test.

‡ Analysis of variance with Levene's test for homogeneity and Welch test.

CFS, Clinical Frailty Scale; CI, confidence interval.

**Fig. 2:**
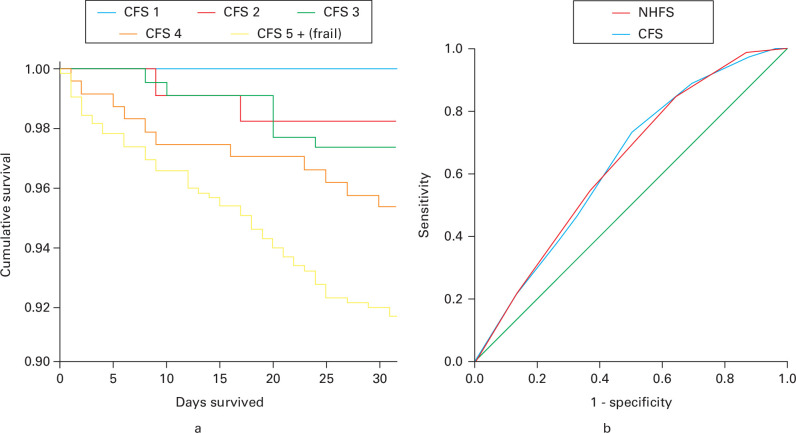
a) Kaplan-Meier survival curve for Clinical Frailty Scale (CFS) and 30-day mortality. b) Receiver operating characteristic curve demonstrating predictive value of CFS and Nottingham Hip Fracture Score (NHFS) with 30-day survival.

Figure 2a illustrates Kaplan-Meier survival curves for CFS frailty scores, where CFS 1 to 4 are presented individually, and CFS 5 to 9 are presented together, in view of the relationship between CFS and 30-day mortality discussed above. The log-rank test demonstrated a significant relationship between CFS and survival (p < 0.001).

Figure 2b illustrates the ROC for the prediction of 30-day mortality for the CFS and the NHFS. This demonstrates that the CFS predicts 30-day mortality similarly to the NHFS (CFS AUC 0.631 (95% CI 0.572 to 0.690); NHFS AUC 0.631 (95% CI 0.571 to 0.690)).

## Discussion

This is the first large-scale study investigating CFS as a predictor of outcomes following fragility fracture of the hip. The CFS is becoming part of routine data collection for hip fractures and often is collected by admitting teams. It is important to note that, given the increasing value placed on frailty, the CFS can be used to distinguish outcomes between those who are frail and not frail, but cannot distinguish differential outcomes between frailty states CFS 5 to 9. The CFS predicted 30-day survival with similar accuracy to the NHFS.

Our study broadly demonstrates a linear relationship between CFS 1 to 4 (patients who are not frail) and surgical outcomes. It could be expected that, as the transition is made to increasing levels of frailty, this linear relationship would continue or demonstrate a steeper gradient. Our study shows that patients who have scores CFS 5 to 9 (mild to severe frailty) have a worse prognosis than those who are not frail. There is no apparent relationship, however, between higher frailty scores and mortality, postoperative complications, and LOS following hip fracture surgery. The exception is in the outcome of institutionalization where there is a linear relationship from CFS 1 to 7. In summary, our results show that frail patients have a poorer outcome than those who are not frail, but in using the CFS we are unable to distinguish differences in outcome for higher frailty levels. It is important to understand this limitation given the increasing popularity of the CFS.

Patients who are frail have reduced physiological reserve, and therefore their ability to ‘bounce back’ following injury may be impaired. There may be a physiological threshold beyond which the effect on outcomes does not further change. This may explain the change of a linear to non-linear relationship from non-frail (CFS 1 to 4) to frail (CFS 5 to 9) in mortality, postoperative complications, and LOS. With the outcome of institutionalization, which is linear from CFS 1 to 7, it is likely that the CFS is being accurately measured in this cohort. The findings of this study would therefore indicate that the CFS does quantify frailty accurately and hence permit prediction of the likelihood of returning to baseline function (as measured by return to independent living). In the acute injury and perisurgery setting, however, frail patients suffer equally poor results whatever the severity of their frailty.

On the other hand, it is possible that our observed lack of increasing complication rates associated with increasing frailty represents a failure of the admitting clinicians to distinguish between the higher frailty grades. This could be due to assessment at the time of a major injury, when more complex background can be hard to ascertain, or lack of expertise. Recently there have been additional tools introduced to help guide more accurate completion of the assessments.^13^

We believe these results to be robust, since the sample size was large and the data were collected prospectively to a high level of completeness. We acknowledge that, due to the limitations of our routine data collection process, we were unable to measure other potential frailty outcomes, such as the incidence of delirium or any residual mobility or functional deficit. We also note our estimate for the LOS was for the acute hospital only, and did not take into account the ‘superspell’, including the time spent in rehabilitation or postoperative care facilities. This will have confounded the observed relationship between CFS and LOS.

Our findings suggest that the CFS may be an imprecise measure of frailty in hip fracture when administered in this way, but it adds to the growing body of evidence indicating the value of assessing frailty in hospital practice in a range of conditions such as Parkinson’s disease,^14^ COVID-19,^15^ and all emergency surgical admissions.^16^ They also confirm previous reports indicating an association between frailty and mortality,^17^ complications,^18^ and LOS^19^ in hip fracture patients. We need to ensure that the role of the orthogeriatrician remains at the centre of care for these patients, especially in the early stages of patient assessment and to guide care planning. For example, many patients with CFS ≥ 5, who are therefore at higher risk of death, complications, and institutionalization, may choose care plans that focus on symptom control rather than life extension. By identifying these patients, discussions about cardiopulmonary resuscitation may be easier if accompanied by robust information about the likelihood of imminent death, whether to start thinking about care homes, moving a bed downstairs, optimizing nutrition, or mental wellbeing. Although we observed a statistically significant association between CFS and LOS, we have to bear in mind that many patients may undergo a period of inpatient rehabilitation or other aftercare prior to discharge home, and this was not taken into account in this study.

While this work demonstrates the value of the principle of the measurement of frailty, and the utility of the CFS tool to do this, the quantification of frailty remains a matter of considerable debate. More sophisticated means of measuring frailty may be needed (for example, by using combinations of simple but valid biomarkers^20^ of organ or system integrity^21^), and further work should be undertaken to explore this. We also have to address the training needed for junior doctors to complete the assessments accurately. Finally, we need to investigate why there is no difference in outcomes between mildly, moderately, and severely frail patients with respect to mortality, postoperative complications, and LOS.

**Take home message**

- The routine use of the Clinical Frailty Scale provides useful indications of information on outcomes for fitter patients presenting with hip fracture.

- However, at higher grades of frailty, further work is needed to understand why patients assessed as being of mild, moderate, and severe frailty do not appear to result in different outcomes.

## References

[1] Baker PN , Salar O , Ollivere BJ , et al. Evolution of the hip fracture population: time to consider the future? A retrospective observational analysis. BMJ Open. 2014;4(4):e004405. 10.1136/bmjopen-2013-004405 PMC3996816 24747789

[2] Kanis JA , Oden A , Johnell O , Jonsson B , de Laet C , Dawson A . The burden of osteoporotic fractures: a method for setting intervention thresholds. Osteoporos Int. 2001;12(5):417–427. 10.1007/s001980170112 11444092

[3] No authors listed . Dashboard Report for All NHFD 2020. National Hip Fracture Database (NHFD), https://www.nhfd.co.uk/20/nhfdcharts.nsf/fmdashboard?readform&year=2020&org=[ALL](date last accessed 17 May 2022).

[4] Rapp K , Rothenbacher D , Magaziner J , et al. Risk of nursing home admission after femoral fracture compared with stroke, myocardial infarction, and pneumonia. J Am Med Dir Assoc. 2015;16(8):715. 10.1016/j.jamda.2015.05.013 26142060

[5] Lawrence VA , Hilsenbeck SG , Noveck H , Poses RM , Carson JL . Medical complications and outcomes after hip fracture repair. Arch Intern Med. 2002;162(18):2053–2057. 10.1001/archinte.162.18.2053 12374513

[6] Krishnan M , Beck S , Havelock W , Eeles E , Hubbard RE , Johansen A . Predicting outcome after hip fracture: using a frailty index to integrate comprehensive geriatric assessment results. Age Ageing. 2014;43(1):122–126. 10.1093/ageing/aft084 23832264

[7] Patel KV , Brennan KL , Brennan ML , Jupiter DC , Shar A , Davis ML . Association of a modified frailty index with mortality after femoral neck fracture in patients aged 60 years and older. Clin Orthop Relat Res. 2014;472(3):1010–1017. 10.1007/s11999-013-3334-7 24166073 PMC3916591

[8] Conroy S , Thompson D , Griffiths S , et al. Improving acute care for older people at scale - the Acute Frailty Network. Acute Med. 2016;15(4):185–192. 28112287

[9] Rockwood K , Song X , MacKnight C , et al. A global clinical measure of fitness and frailty in elderly people. CMAJ. 2005;173(5):489–495. 10.1503/cmaj.050051 16129869 PMC1188185

[10] Moppett IK , Parker M , Griffiths R , Bowers T , White SM , Moran CG . Nottingham Hip Fracture Score: longitudinal and multi-assessment. Br J Anaesth. 2012;109(4):546–550. 10.1093/bja/aes187 22728204

[11] Parker MJ , Currie CT , Mountain JA , Thorngren K-G . Standardised Audit of Hip Fracture in Europe (SAHFE). HIP Int. 2018;8(1):10–15. 10.1177/112070009800800106

[12] Hodkinson HM . Evaluation of a mental test score for assessment of mental impairment in the elderly. Age Ageing. 1972;1(4):233–238. 4669880 10.1093/ageing/1.4.233

[13] Rockwood K , Fay S , Theou O , Dykes L . Top tips to help you use the Clinical Frailty Scale. 2020. https://cdn.dal.ca/content/dam/dalhousie/pdf/sites/gmr/CFS-Top-Tips_2020Jun05_EN_Letter.pdf(date last accessed 17 May 2022).

[14] Torsney KM , Romero-Ortuno R . The Clinical Frailty Scale predicts inpatient mortality in older hospitalised patients with idiopathic Parkinson’s disease. J R Coll Physicians Edinb. 2018;48(2):103–107. 10.4997/JRCPE.2018.201 29992197

[15] Hewitt J , Carter B , Vilches-Moraga A , et al. The effect of frailty on survival in patients with COVID-19 (COPE): a multicentre, European, observational cohort study. Lancet Public Health. 2020;5(8):e444–e451. 10.1016/S2468-2667(20)30146-8 32619408 PMC7326416

[16] Hewitt J , Carter B , McCarthy K , et al. Frailty predicts mortality in all emergency surgical admissions regardless of age. An observational study. Age Ageing. 2019;48(3):388–394. 10.1093/ageing/afy217 30778528

[17] Narula S , Lawless A , D’Alessandro P , Jones CW , Yates P , Seymour H . Clinical Frailty Scale is a good predictor of mortality after proximal femur fracture: A cohort study of 30-day and one-year mortality. Bone Jt Open. 2020;1(8):443–449. 10.1302/2633-1462.18.BJO-2020-0089.R1 33215137 PMC7667224

[18] Chan S , Wong EKC , Ward SE , Kuan D , Wong CL . The predictive value of the Clinical Frailty Scale on discharge destination and complications in older hip fracture patients. J Orthop Trauma. 2019;33(10):497–502. 10.1097/BOT.0000000000001518 31188261

[19] Yoo J , Lee JS , Kim S , et al. Length of hospital stay after hip fracture surgery and 1-year mortality. Osteoporos Int. 2019;30(1):145–153. 10.1007/s00198-018-4747-7 30361752

[20] Soysal P , Stubbs B , Lucato P , et al. Inflammation and frailty in the elderly: A systematic review and meta-analysis. Ageing Res Rev. 2016;31:1–8. 10.1016/j.arr.2016.08.006 27592340

[21] El Assar M , Angulo J , Rodríguez-Mañas L . Frailty as a phenotypic manifestation of underlying oxidative stress. Free Radic Biol Med. 2020;149:72–77. 10.1016/j.freeradbiomed.2019.08.011 31422077

